# Dynamics of SAS-I mediated H4 K16 acetylation during DNA replication in yeast

**DOI:** 10.1371/journal.pone.0251660

**Published:** 2021-05-20

**Authors:** Mark Boltengagen, Anke Samel-Pommerencke, David Fechtig, Ann E. Ehrenhofer-Murray

**Affiliations:** Institut für Biologie, Humboldt-Universität zu Berlin, Berlin, Germany; Texas A&M University, UNITED STATES

## Abstract

The acetylation of H4 lysine 16 (H4 K16Ac) in *Saccharomyces cerevisiae* counteracts the binding of the heterochromatin complex SIR to chromatin and inhibits gene silencing. Contrary to other histone acetylation marks, the H4 K16Ac level is high on genes with low transcription, whereas highly transcribed genes show low H4 K16Ac. Approximately 60% of cellular H4 K16Ac in *S*. *cerevisiae* is provided by the SAS-I complex, which consists of the MYST-family acetyltransferase Sas2, Sas4 and Sas5. The absence of SAS-I causes inappropriate spreading of the SIR complex and gene silencing in subtelomeric regions. Here, we investigated the genome-wide dynamics of SAS-I dependent H4 K16Ac during DNA replication. Replication is highly disruptive to chromatin and histone marks, since histones are removed to allow progression of the replication fork, and chromatin is reformed with old and new histones after fork passage. We found that H4 K16Ac appears in chromatin immediately upon replication. Importantly, this increase depends on the presence of functional SAS-I complex. Moreover, the appearance of H4 K16Ac is delayed in genes that are strongly transcribed. This indicates that transcription counteracts SAS-I-mediated H4 K16 acetylation, thus “sculpting” histone modification marks at the time of replication. We furthermore investigated which acetyltransferase acts redundantly with SAS-I to acetylate H4 K16Ac. *esa1Δ sds3Δ* cells, which were also *sas2Δ sir3Δ* in order to maintain viability, contained no detectable H4 K16Ac, showing that Esa1 and Sas2 are redundant for cellular H4 K16 acetylation. Furthermore, *esa1Δ sds3Δ sas2Δ sir3Δ* showed a more pronounced growth defect compared to the already defective *esa1Δ sds3Δ sir3Δ*. This indicates that SAS-I has cellular functions beyond preventing the spreading of heterochromatin.

## Introduction

The acetylation of lysine residues in histones plays an important role in regulating gene expression in eukaryotes. Many histone acetyltransferase (HAT) complexes are recruited to gene promoters via interaction with DNA-binding transcriptional activators, and they acetylate histone lysines, which enhances gene activation [1]. In contrast to this, acetylation by the SAS-I (“*something about silencing”*) HAT complex in *Saccharomyces cerevisiae* is anti-correlated with transcription [2]. SAS-I consists of the MYST family HAT Sas2, the cullin domain protein Sas4 and the YEATS protein Sas5 [3, 4], and it performs approx. 60% of cellular acetylation of lysine 16 on histone H4 (H4 K16Ac) [5, 6]. Interestingly, in contrast to other sites of histone acetylation, H4 K16Ac levels are low in promoters, but higher within the open reading frame of yeast genes. Furthermore, H4 K16Ac levels are inversely correlated with the level of transcription: Highly expressed genes have low H4 K16Ac in the body of the gene, and H4 K16Ac increases in a SAS-I dependent manner as a gene is switched off [2, 7]. Also, H4 K16Ac is low in regions of repressed chromatin, e.g. at the telomeres and the silent mating-type loci *HML* and *HMR* [4, 8]. H4 K16Ac specifically inhibits the binding of the repressive *silent information regulator* complex (SIR) to these regions by restricting the interaction of the bromo-adjacent homology domain (BAH) of Sir3 to the nucleosome [9]. In the absence of SAS-I, H4 K16Ac levels are reduced, which leads to the spreading of the SIR complex from the telomeres into subtelomeric regions and to inappropriate silencing of genes that are located in these regions. While spreading of the SIR heterochromatin complex in *sas2Δ* cells is not a lethal event, the combination of *sas2Δ* with loss of the histone deacetylase complex Rpd3L, which also enhances SIR spreading, causes lethality [10]. Thus, the major cellular function of the SAS-I complex is to provide genome-wide H4 K16Ac and to prevent inappropriate gene silencing by the SIR complex.

In contrast to other HAT complexes, SAS-I is not recruited to chromatin by binding to transcriptional activators. Rather, SAS-I interacts with two histone chaperones, CAF-I and Asf1 [3, 4]. CAF-I is required for chromatin assembly after DNA replication [11]. As the replisome proceeds through chromatin, the nucleosomes are partially disassembled in front of the replication fork. Parental histones are subsequently transferred to the two daughter strands by Mcm2, which is part of the MCM helicase complex [12]. CAF-I then adds the full H3/ H4 complement by depositing newly synthesized H3/ H4 onto chromatin. In this process, the Asf1 histone chaperone binds H3/ H4 dimers and delivers them to CAF-I for chromatin assembly. The interaction of SAS-I with CAF-I and Asf1 suggests that it establishes H4 K16Ac on fresh histones as they are deposited by CAF-I and Asf1 shortly after DNA replication.

While SAS-I is the major H4 K16 acetyltransferase in yeast, *sas2Δ* cells still have approx. 40% of wild-type levels of H4 K16Ac, raising the question which HAT (or HATs) is responsible for the remainder of the H4 K16 acetylation. One candidate is the HAT Esa1, which, as a member of the MYST family of HATs, is a homolog of Sas2 [13]. Esa1 is the only essential HAT in *S*. *cerevisiae* and is the catalytic subunit of the larger HAT complex NuA4 and the smaller piccolo NuA4. Esa1/ NuA4 acetylates H4 on K5, K8, K12 and, to a lesser degree, K16 [14–16] as well as H2A and H2B [17], and it is required for the activation of ribosomal protein genes [18].

In this study, we investigated the genome-wide dynamics of H4 K16Ac during DNA replication and its dependence on SAS-I. We observed that this mark appears in chromatin at the time of replication, and that the replication-dependent increase of H4 K16Ac fully depends on the SAS-I complex. Furthermore, the deposition of H4 K16Ac was delayed in genes with higher transcription as compared to genes with low transcription, indicating that histone turnover during transcription counteracts replication-dependent H4 K16Ac deposition by SAS-I. Furthermore, we found that Esa1 is responsible for the vast majority of H4 K16Ac in the absence of SAS-I, since H4 K16Ac was undetectable in cells lacking both Sas2 and Esa1.

## Materials and methods

### *S*. *cerevisiae* strains and methods

Yeast strains used in this study are described in Table 1. Yeasts were grown and manipulated using standard genetic techniques [19]. Auxin was added to the growth medium at a concentration of 1 mM where indicated. Cells were arrested in S-phase with hydroxyurea (HU, 0.2 M). After release from HU arrest, cell cycle progression was monitored by FACS analysis [20]. For this purpose, the cells were washed with 800μl of 50 mM sodium citrate (pH 7.2) and incubated with 500 μl RNase A solution (0.25mg/ml in 50 mM sodium citrate (pH7.2)) overnight at 37°C. The cells were subsequently incubated with 20μl of 20mg/ml proteinase K for 2 hours at 49°C, sonicated briefly and stained with 500μl Sytox Green solution (4μM Sytox Green in 50 mM sodium citrate (pH 5.2)). FACS analysis was performed with a BD Accuri flow cytometer.

**Table 1:** *S*. *cerevisiae* strains used in this study.

Strain	Genotype	Source*
AEY2	*MAT***a** *ade2-101 his3-11*,*15 trp1-1 leu2-3*,*112 ura3-1 can1-100* (W303)	
AEY4	*MAT***a** *ADE2 his3-11*,*15 trp1-1 leu2-3*,*112 ura3-1 lys2Δ can1-100*	
AEY17	*MATα ADE2 his3-11*,*15 trp1-1 leu2-3*,*112 ura3-1 can1-100 lys2Δ sir3Δ*∷*TRP1*	
AEY266	AEY2 *sas2Δ*∷*TRP1*	
AEY346	*MAT***a** *ade2-101 his3-11*,*15 trp1-1 leu2-3*,*112 ura3-1 can1-100 sir1Δ*∷*LEU2 sas2Δ*∷*TRP1*	
AEY3756	*MAT***a** *ade2-101 his3-11*,*15 TRP1 leu2-3*,*112 ura3-1 can1-100 LYS sds3Δ*∷*kanMX*	
AEY4658	*MATα ADE2 his3-11*,*15 trp1-1 leu2-3*,*112 ura3-1 can1-100 lys2Δ sir3Δ*∷*HIS3 sas2Δ*∷*TRP1*	
AEY5945	*MAT***a** *ade2-101 his3-11*,*15 trp1-1 leu2-3*,*112 ura3-1 can1-100 ura3-1*∷*AHD1-OsTIR1-9myc (URA3)* (BY25598)	Yeast Genetic Resource Center ** [21]
AEY6141	*MAT***a** *ura3-1*∷*ADH1-OsTIR1 (URA3) Sas4-3xmini-AID-9xMYC*∷*KanMX ade2-1 his3-11*,*15 trp1-1 leu2-3*,*112 can1-100*	
AEY6127	*MAT***a** *ura3-1*∷*ADH1-OsTIR1 (URA3) Sas4-3xmini-AID-9xMYC*∷*KanMX sir1Δ*∷*LEU2 ade2-1 his3-11*,*15 trp1-1 leu2-3*,*112 can1-100*	
AEY6468	*MATa ade2-101 his3-11*,*15 TRP1 leu2-3*,*112 ura3-1 can1-100 LYS sds3Δ*∷*KanMX sir3Δ*∷*NatMX*	
AEY6523	*MATα ade2-101 trp1-1 leu2-3*,*112 ura3-1 can1-100 LYS sds3Δ*∷*KanMX sas2Δ*∷*HIS3 sir3Δ*∷*NatMX*	
AEY6597	*MATa ADE2 trp1-1 leu2-3*,*112 ura3-1 can1-100 lys2Δ sds3Δ*∷*KanMX esa1Δ*∷*UraMX*	
AEY6605	*MATa ade2-101 his3-11*,*15 TRP1 leu2-3*,*112 ura3-1 can1-100 LYS2 sds3Δ*∷*KanMX sir3Δ*∷*NatMX esa1Δ*∷*UraMX*	
AEY6652	*MAT***a** or *MATα ade2-101 trp1-1 leu2-3*,*112 ura3-1 can1-100 LYS sds3Δ*∷*KanMX sas2Δ*∷*HIS3 sir3Δ*∷*NatMX esa1Δ*∷*klTRP1*	

* Unless indicated otherwise, strains are derivatives of AEY2 and were constructed in the course of this study or are from the laboratory collection

** Strain was provided by NBRP of the MEXT, Japan.

Deletions of chromosomal genes were performed using the integration of knockout cassettes [22]. *SAS4-AID* was generated by integration of *3xmini-AID-9xmyc*∷*kanMX* at the 3’ end of *SAS4*.

The *sas2Δ esa1Δ sds3Δ sir3Δ* strain was obtained by deleting *ESA1* (*esa1Δ*∷*KlTRP1*) in a diploid of AEY6523 and AEY4 containing a *URA3*-marked plasmid carrying *SAS2*. The resulting *esa1Δ/ ESA1* heterozygous diploid was sporulated, and a *sas2Δ esa1Δ sds3Δ sir3Δ* segregant carrying p*URA3*-*SAS2* was selected. The segregant was subsequently streaked on medium with 5-fluoro-orotic acid (5-FOA) in order to select for isolates that had lost the p*URA3*-*SAS2* plasmid, which resulted in strain AEY6652.

Semi-quantitative mating assays were performed by generating serial dilutions (1:10, start OD_600_ of one) of the respective strain in a micro-titer dish. For the growth control, cells were transferred to agar plates using a replica tool. An equal volume of the mating tester strain (suspension of 10 OD_600_ per milliliter) was then added to the strain in the microtiter well, and a replica of this mixture was transferred to a plate selective for the growth of diploids. Plates were incubated for 2–3 d at 30°C.

### Chromatin immunoprecipitation

ChIP of H4 K16Ac and H4 and quantitative real-time PCR were performed essentially as previously described [23], with some modifications. Yeast cells were grown to an OD600 of 0.6–0.9 at 30°C and cross-linked with 1% formaldehyde for 10 minutes with shaking at room temperature. The cross-linking reaction was stopped with 125 mM glycine for 5 min. Cells were harvested by centrifugation (3 minutes, 3500 rcf) and washed twice in 1× TBS. Pelleted cells were washed with 1 ml spheroplasting buffer (1 M sorbitol, 100 mM KPO_4_ [pH 7.5] and 30 mM β-mercaptoethanol), pelleted again (5 minutes, 3500 rcf) and resuspended in 0.5 ml spheroplasting buffer with 40 unit/ml zymolyase and cells were spheroplasted for 30 minutes at 30°C with shaking at 350 rpm. Spheroplasts were pelleted by centrifugation (5 minutes, 3500 rcf) and washed twice with spheroplasting buffer. Pelleted spheroplasts were resuspended in MNase buffer (50 mM NaCl, 10 mM Tris pH 7.5, 5 mM MgCl_2_, 1 mM CaCl_2_, 0.075% NP-40, 1 mM β-mercaptoethanol, 0.5 mM spermidine). Chromatin was then fragmented by digestion with 40 units of micrococcal nuclease (MNase) for 25 minutes at 30°C. The reaction was stopped by adding 10 mM EDTA and incubating for 5 minutes at 4°C. The lysate was diluted with 1 ml of 1.5x lysis buffer (1x lysis buffer: 50 mM HEPES (pH7.5), 1 mM EDTA, 140 mM NaCl, 1% (v/v) Triton X-100 and 0.1% sodium deoxycholate) containing protease inhibitors) and centrifuged for 10 min (14,000 rpm) at 4°C. 500 μl of the supernatant was used for immunoprecipitation and 200 μl for the input control.

Aliquots were precleared with Dynabeads protein G (Thermo Fisher) for 2 h at 4°C and incubated over night with 4 μl α-H4 K16Ac antibody (Millipore 07–329) or 4 μl α-H4 antibody (abcam ab7311). After incubation, the lysates were treated with Dynabeads Protein G for 2 h at 4°C. The immunoprecipitates were washed with 1 ml of the following buffers (ice-cold): 1) low salt solution (0.1% (v/v) SDS, 1% (v/v) Triton X-100, 2 mM EDTA, 20 mM Tris (pH 8.1) 150 mM NaCl); 2) high salt solution (0.1% (v/v) SDS, 1% Triton (v/v) X-100, 2 mM EDTA, 20 mM Tris (pH 8.1) 500 mM NaCl); 3) LiCl buffer (0.25 M LiCl, 1% (v/v) Nonidet P-40, 1% (w/v) sodium deoxycholate, 1 mM EDTA, 10 mM Tris pH 8.1), twice 1× TE. The samples and the input DNA were subsequently treated with elution buffer (1% (v/v) SDS, 0.1 M NaHCO_3_), incubated for 10 minutes at 99°C to reverse cross-linking and incubated 1 h at 37°C with 1 μl RNAse (10 mg/ml) and 1 h with proteinase K (Roche, 1 μl of 10 mg/ml). DNA samples were extracted with Chelex 100 resin (Bio-Rad) and analysed by real-time PCR. Primer sequences are available upon request.

### Cell synchronization in S-phase for ChIP-seq

Yeast cells were synchronized in S-phase using HU as follows. Cells were freshly grown at 30°C in YPD to obtain an exponentially growing culture of OD600 0.2–0.5 (900 ml). HU was subsequently added to 0.2M, cells were incubated for 1.5h, and synchronization was followed by FACS. A sample of 30 OD was collected (timepoint “0”). The culture was then split, auxin (0.1 mM) or ethanol (control) was added, and the cells were incubated for a further 30 minutes. After collection of a further 30 OD sample, the cells were collected by centrifugation, washed with pre-warmed YPD and resuspended in 800 ml of pre-warmed YPD. 30 OD samples were collected every 10 minutes for 60 minutes and processed in parallel for ChIP-seq.

### ChIP-seq

Chromatin immunoprecipitation for high-throughput sequencing was performed as above with some modifications. In cell synchronization experiments, 30 OD of cells were used per sample. Samples were cross-linked with 1% formaldehyde for 5 minutes at 30°C and shaking. Cross-linking was stopped by incubating cells with 125 mM glycine for 5 minutes at RT. Cells were then washed with TBS, and cell pellets were snap frozen at -80°C. Cells were resuspended in spheroplasting buffer, and spheroplasts were obtained by zymolyase digestion (40 U/ml zymolyase, 40 min at 30°C). Pellets were washed once with spheroplasting buffer, speroplasts were resuspended in 500 μl of MNase buffer, and chromatin was digested with micrococcal nuclease (25 min, 30°C). The digestion was stopped by adding EDTA to 10 mM and incubating at 4°C. The MNase-digested chromatin was diluted with 1.5x lysis buffer, and the lysate was cleared by centrifugation for 10 minutes (14,000 rpm, 4°C). As a spike-in control, 3–5% of sheared chromatin from *Schizosaccharomyces pombe* (see below) was added. 2 x 500 μl of the lysates were transferred to low protein binding Eppendorf tubes, 200 μl were used for input, and the remaining 250 μl were used to control for chromatin digestion.

Samples were pre-cleared by incubation with 30 μl beads (Dynabeads Protein G, Invitrogen, 2 h 4°C). The pre-cleared supernatant was incubated over night (4°C) with 4 μl of α-H4 K16Ac antibody (Millipore 05–1232). Immunoprecipitates were collected by incubation with 60 μl beads for 2 h at 4°C. Beads were then washed as above, suspended in 200 μl elution buffer (1% SDS, 0.1 M NaHCO_3_), and chromatin was eluted from the beads for 1 h at 65°C. 8 μl 5M NaCl was added to the immunoprecipitated samples as well as to the input, and samples were reverse cross-linked over night at 65°C. After RNAse digestion (1 h 37°C, 1 μl of 10 mg/ ml) and Proteinase K digestion (2 μl of 10 mg/ml Proteinase K, 10 μl 0.5 M EDTA, 20 μl Tris-HCl pH 6.5, 1 h 49°C, shaking), 5 μl of NaAc pH 5.5 and 1ml of Qiagen buffer PB were added, and DNA was collected using Qiagen PCR cleanup columns (QIAquick PCR Purification Kit, Qiagen). DNA was eluted with 30μl of Qiagen EB buffer. ChIP libraries were indexed (NEXTflex qChIP-Seq Library Prep kit, Perkin Elmer), pooled and sequenced on an Illumina NextSeq500 machine.

### Preparation of *S*. *pombe* chromatin for spike-in

*S*. *pombe* strain AEP1 (*h*^*-*^ *leu1-32 ura4-D18 his3-D3*) was grown in YES medium to 1 OD/ml. 100 OD cells were collected, washed twice with TBS buffer and suspended in 1 ml spheroplasting buffer. Cells were treated with zymolyase T-100 for 30 min and washed twice with spheroplasting buffer. Spheroplasts were then lysed with 1 ml lysis buffer by pipetting up and down. Samples were sonicated for 15 min (30 sec ON, 30 sec OFF, high settings) with a Bioruptor and cleared by centrifugation at 14000 rpm for 10 min. 200 μl aliquots of the supernatant were snap-frozen in liquid nitrogen and added to MNase-digested *S*. *cerevisiae* samples at 3–5% of the total sample. Lysates were then processed for ChIP as described above.

### Data processing and analysis of ChIP-seq data

Sequence reads were aligned to the genomes of *S*. *cerevisiae* (SacCer3) and *S*. *pombe* using Bowtie 2. Genomic tracks were generated using MACS 2.0 [24] on mapped reads from the ChIP-seq samples compared to their respective input control. Tracks were subsequently normalized by a factor *k* using the *S*. *pombe* spike-in: *k* = (IP_sc_/IP_sp_)*(IN_sp_/IN_sc_), where IP_sc_, IP_sp_, IN_sp_ and IN_sc_ are the reads in IP and input (IN) in spike-in (*S*. *pombe*, sp) and sample (*S*. *cerevisiae*, sc), respectively.

For the replication time series, profiles were further normalized using a polynomial fit of the genome-wide averages of H4 K16Ac.

### Plotting

For heat maps of H4 K16Ac during replication (Figs 2D and 3), normalization was done by log_2_-transforming the 15-kB bin average (moving average 2 kB) and then subtracting the average signal of the bin along the time-course, as described previously [25]. For plots of H4 K16Ac around genomic features (ARS, TSS, Fig 4), the mean level of acetylation at each base around that feature was calculated for a given group of genes or genomic regions (ARS). For TSS plots, genes were ranked by their expression level in cells arrested in HU for 2 hours, as determined by RNA-seq. Origins of replication (ARS) were grouped by their replication time [26]. For plots in Fig 4D, 4F, the mean H4 K16Ac level of 2000 bp upstream and downstream of the ARS elements, but excluding the nucleosome-depleted region (ARS +/- 250 bp), was computed. All scripts are available in the R package MNuc (https://github.com/suvarzz/MNuc).

### RNA extraction and sequencing

RNA was extracted from a culture of yeast cells arrested for 2 hours in HU, and RNA was extracted as follows. 30 OD of cells were collected, and the cell pellet was resuspended in 5 ml of peqGOLD TriFast (VWR). 3 ml of acid-washed glass beads were added, and cells were lysed by vortexing for 5 minutes. The lysate was transferred to a phase trap tube, 5 ml chloroform was added, and the sample was centrifuged at 1500 g for 5 minutes. The supernatant was mixed with 5 ml isopropanol, RNA precipitated over night at -20°C, and collected by centrifugation at 10,000 g for 30 minutes. The pellet was washed with 70% ethanol and resuspended in DEPC-treated H_2_O. For RNA-seq, fragmented, poly(A)-selected RNA of approx. 200 bp size were reverse transcribed using barcoded poly(T) primers. cDNA was amplified and sequenced on an Illumina NextSeq500 machine. The RNA sequence data was analysed using the Salmon tool [27], and transcripts were sorted by transcription level.

### Yeast protein extracts and Western blotting

For western blot analysis, samples of 10 OD of cells were harvested, washed once with TBS and resuspended in 120 μl lysis buffer (50 mM Tris pH 8, 300 mM NaCl, 1% NP-40, 0.5% sodium-deoxycholate, 0.1% SDS, 10 mM sodium fluoride 0.5 mM EDTA, 10% glycerol, 1.5 μl sodium-butyrate (5 mM) and protease inhibitors). The proteins were extracted by vortexing 5 minutes at 4°C with acid-washed glass beads. 40 μl of 4xLämmli buffer was added to each sample, and samples were heated for 3 min to 95°C. Protein amounts equivalent to 1 OD cells were separated on SDS-PAGE gels (bottom: 15%, top 10%) and analysed by Western blotting. Antibodies used for western blotting were α-H4 K16Ac (Millipore 05–1232), α-myc (Sigma M4439) and α-GAPDH (loading control, Abcam ab9385). The immunoblots were imaged on a Bio-Rad imaging system.

### Data accessibility

ChIP-seq data was deposited in the SRA archives (accession No. PRJNA511477, https://www.ncbi.nlm.nih.gov/bioproject/PRJNA511477/).

## Results

### Construction of *SAS4-AID*

Our previous work has shown that approx. 60% of bulk acetylation of H4 K16 is mediated by the SAS-I histone acetyltransferase complex, and it is deposited in chromatin during the S-phase of the cell cycle [7]. Here, we sought to measure the dynamics of H4 K16Ac in chromatin during S-phase in the presence or absence of the SAS-I complex. We have previously used a version of Sas2 with a temperature-sensitive N-terminal degron [7], but this construct requires changes in temperature and carbon source to inactivate Sas2, which both are conditions that cause unwanted physiological perturbations to the cells. We therefore sought to obtain a regulated version of SAS-I that does not require major changes in media or temperature.

**Fig 1:**
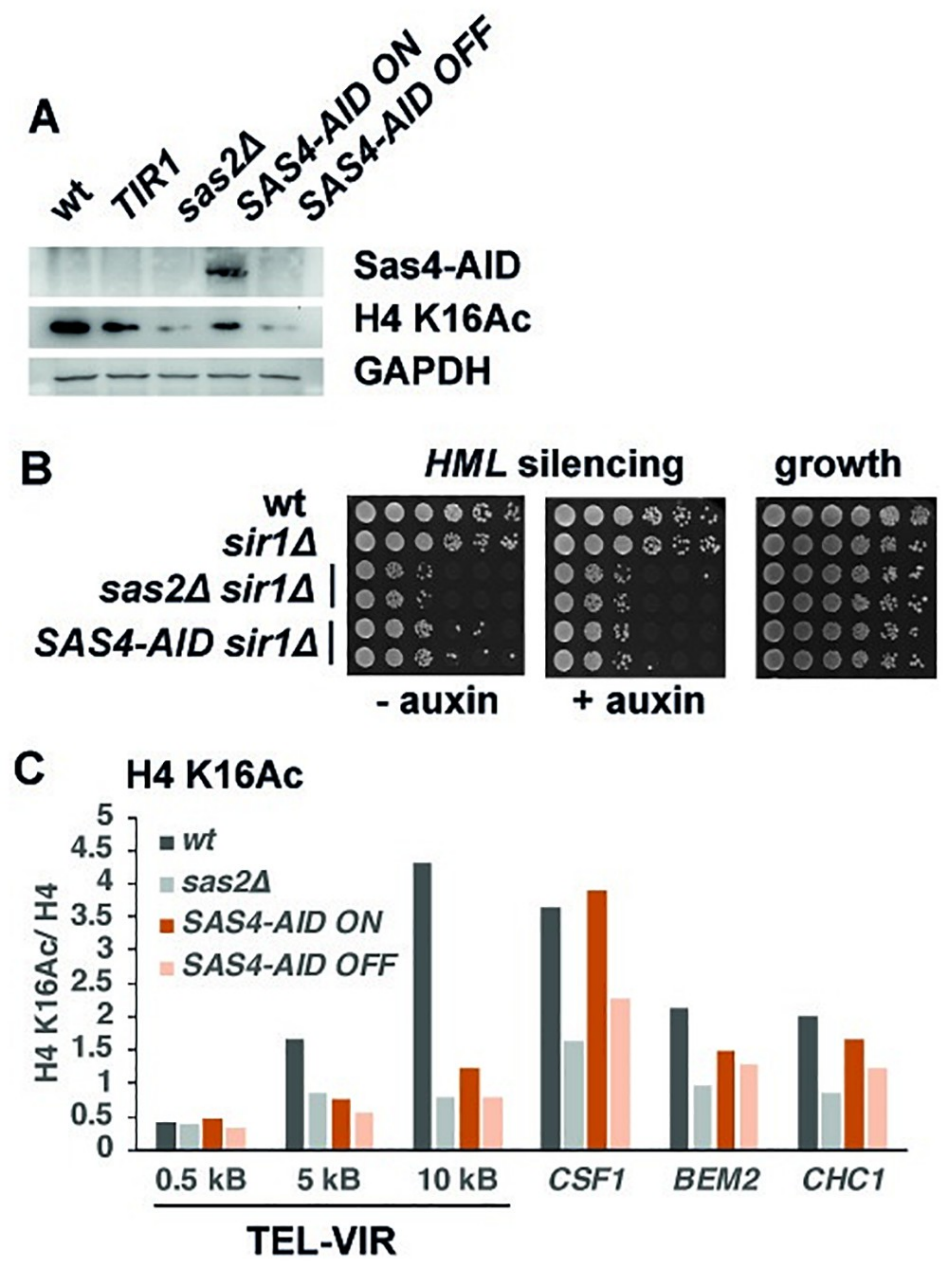
Establishment of an auxin-regulated version of the SAS-I component *SAS4*. (A) *SAS4-AID (SAS4-AID)* downregulation by addition of auxin (OFF) caused a reduction of bulk H4 K16Ac. Top, Detection of myc-tagged Sas4-miniAID by Western blotting with an α-myc antibody; middle, α-H4 K16Ac; bottom, GAPDH (loading control). (B) Sas4-AID provided Sas4 function in *HML* silencing. Semi-quantitative mating of the indicated *MAT***a** strains in the presence or absence of auxin is shown. (C) Reduction of H4 K16Ac levels by downregulation of Sas4-AID at subtelomeric regions on the right arm of telomere VI (TEL-VIR) and selected genes, as measured by ChIP. Means of two independent biological replicates of H4 K16Ac relative to H4 are shown.

To this end, we constructed a version of Sas4 with an auxin-inducible degron (AID). In the AID system, the plant hormone auxin (indole-3-acetic acid, IAA) induces an interaction between the degron and the plant F-box protein TIR1 that is heterologously expressed in yeast. This leads to the ubiquitination and proteasomal degradation of the degron-carrying protein [21]. We used a minimal version of AID (3xminiAID) that contains a 9xmyc epitope tag to allow detection of the fusion protein. *SAS4-miniAID* (hereafter termed *SAS4-AID*) was readily detectable in the absence of auxin, but was undetectable in its presence (Fig 1A). Furthermore, down-regulation of *SAS4-AID* caused a reduction in bulk H4 K16Ac levels to a similar degree as in *sas2Δ* cells (Fig 1A), showing that the addition of auxin caused strong degradation of the Sas4-miniAID fusion protein.

We further tested the functionality and sensitivity of Sas4-AID to auxin by determining its effect on *HML* silencing. The absence of the SAS-I complex (*sas2Δ* or *sas4Δ*) causes a defect in *HML* silencing in *sir1Δ* cells [28–31]. Importantly, *HML* silencing in *SAS4-AID sir1Δ* cells was reduced in the presence of auxin compared to without auxin, showing that Sas4-AID is sensitive to auxin-mediated degradation (Fig 1B). However, silencing in the absence of auxin was not as strong as in *sir1Δ*, indicating that Sas4-AID had reduced function compared to wild-type Sas4. Of note, *SAS2-miniAID* and *SAS5-miniAID* versions were non-functional.

Next to the loss of bulk H4 K16Ac in auxin-treated *SAS4-AID* cells, we also tested for the loss of chromatin-bound H4 K16Ac by chromatin-immunoprecipitation (ChIP). The SAS-I complex is responsible for H4 K16Ac in subtelomeric regions as well as in the body of genes with a low level of transcription, and the absence of Sas2 (*sas2Δ*) causes a loss of H4 K16Ac in these regions [2]. In agreement with repressed *SAS4-AID* causing a loss of SAS-I function, H4 K16Ac was reduced at distances of 5 and 10 kb from the telomere end of chromosome VI-R in the presence of auxin, and it was reduced in the open reading frame of the genes *CSF1*, *BEM2* and *CHC1* in the presence compared to the absence of auxin (Fig 1C). These genes were analyzed, because our earlier analysis had shown that they have a pronounced loss of H4 K16Ac in *sas2Δ* [2]. However, H4 K16Ac levels at most loci were not as high in *SAS4-AID* as in wild-type (wt) cells, again indicating reduced function of Sas4-AID.

### SAS-I mediates the increase of H4 K16Ac during DNA replication

**Fig 2:**
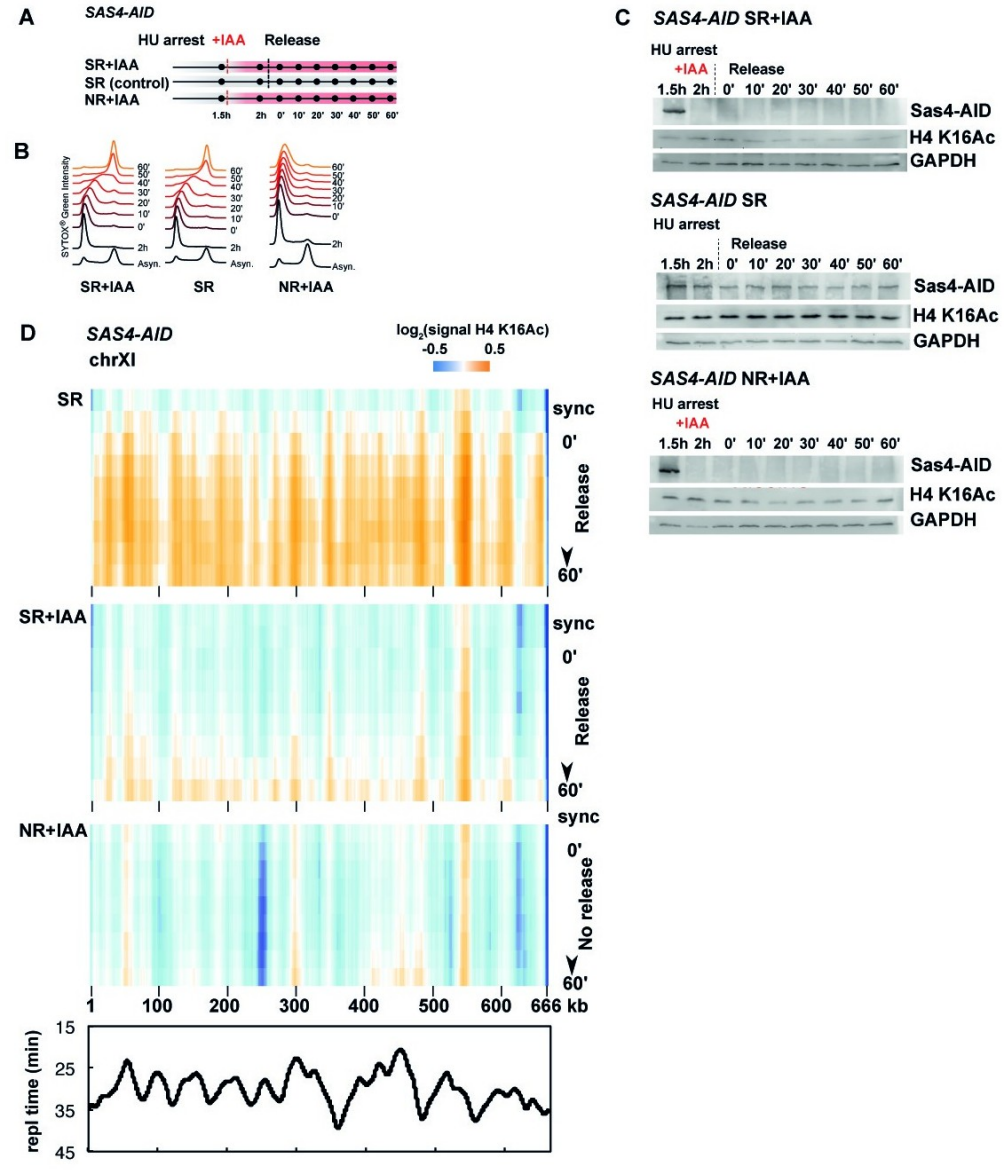
Replication-coupled acetylation of H4 K16 depends on the SAS-I histone acetyltransferase complex. (A) Experimental set-up to measure the dynamics of H4 K16Ac during S-phase. Cells were arrested in S-phase with HU for 1.5 hours; Sas4-AID was subsequently depleted by the addition of auxin (+IAA) for 30 minutes in the samples SR+IAA and NR+IAA (the solvent ethanol is added to the control experiment, SR). Cells of the SR+IAA and SR samples were then collected by centrifugation and resuspended in medium lacking HU. Samples were collected in 10 minute intervals and processed for FACS (B), Western blotting (C) and ChIP-seq (D). (C) Monitoring the depletion of Sas4-miniAID and H4 K16Ac in the synchronization/ release experiments. Blots were performed as in Fig 1A. Sync, synchronization. (D) Replication-associated dynamics of H4 K16Ac. The abundance of H4 K16Ac along chromosome XI is shown. Each plot gives the signal along the chromosome (horizontal) at the different times (vertical) relative to the average of the entire time-course. SR, Sas4-AID is on; SR+IAA, Sas4-AID is off; bottom, NR+AID, Sas4-AID is off, but cells were maintained in S-phase (“no release”, see A). Bottom, replication profile of chromosome XI as previously determined [26]. Replication time is plotted as a function of the coordinate.

We next sought to determine how H4 K16Ac changes across the genome during DNA replication in S-phase, and how this acetylation depends on the presence of the SAS-I complex. The following synchronization/ release (SR) protocol was chosen for this purpose (Fig 2A) [25]: *SAS4-AID* cells were arrested in S-phase by adding hydroxyurea (HU) for 1.5 hours. Sas4-AID was subsequently depleted (or not) by adding auxin (synchronization/ release with auxin, SR+IAA) or solvent (SR (control)) to the HU-arrested culture for 30 minutes. Cells were then released into S-phase, and samples were collected every 10 minutes for 60 minutes for profiling of H4 K16Ac by ChIP combined with high-throughput sequencing (ChIP-seq). To control for the effect of DNA replication on H4 K16Ac levels in the absence of Sas4, HU-arrested, Sas4-depleted cells were maintained in HU (“non-released with auxin”, NR+IAA), and samples for ChIP-seq were collected across the same time-course as for the released cells. The synchronization progression was verified by measuring the DNA content of the cells using fluorescence-activated cell sorting (FACS, Fig 2B), which showed that the cells were successfully arrested with HU, and that they progressed with similar speed through S-phase after HU release, regardless of whether Sas4 was on (SR) or off (SR+IAA). Furthermore, Western blotting confirmed the efficient depletion of Sas4-AID upon IAA treatment (Fig 2C). A mild reduction of bulk H4 K16Ac levels upon Sas4-AID depletion was observed (Fig 2C, top, bottom), whereas H4 K16Ac levels remained constant after HU release in the presence of Sas4-AID (Fig 2C, middle).

**Fig 3:**
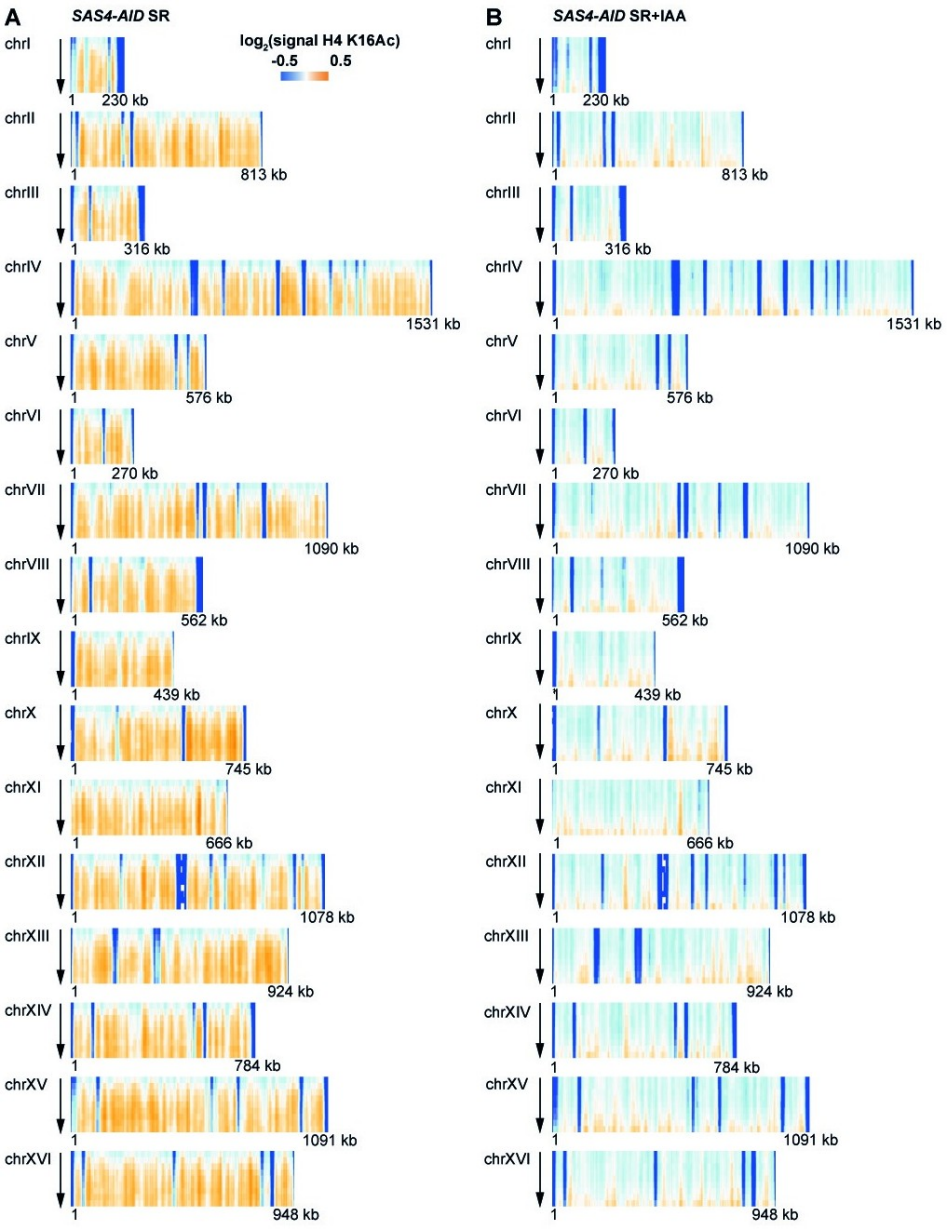
Genome-wide dynamics of H4 K16Ac during replication. H4 K16Ac levels during replication were measured in the presence (A, SR) and absence (B, SR+IAA) of Sas4. Data is represented as in Fig 2D.

We next examined the spatial pattern of H4 K16Ac by plotting its abundance along chromosomes (x-axis) relative to the time of progression through S-phase. Importantly, in the presence of Sas4-AID (SR), we observed a triangle-shaped pattern of H4 K16Ac (Figs 2D and 3A). That is, acetylation increased at defined genomic regions along the chromosome at early time-points. These sites reflect the position of early replication origins (Fig 2D, bottom). At later times, as cells progress through S-phase, the H4 K16Ac profile broadens, indicating that the modification is deposited during S-phase concomitantly with DNA replication [25].

Interestingly, in Sas4-AID-depleted cells, there was a strongly reduced deposition of H4 K16Ac at the sites of DNA replication. Only low levels of H4 K16Ac were apparent at later time-points in S-phase (Figs 2D and 3B). Thus, despite the fact that the Sas4-AID depletion only mildly reduced bulk H4 K16Ac levels, the deposition of H4 K16Ac in chromatin was severely disturbed in the absence of active Sas4.

A further comparison of the global H4 K16Ac pattern of replicating, Sas4-depleted cells to the pattern in non-released cells (NR+IAA) showed that H4 K16Ac levels remained constant across the time course until late time-points, where there was a mild increase in H4 K16Ac at some replication origins (Fig 2D, S1 Fig). This may be due to a minority of cells that escape the HU arrest and proceed through DNA replication after prolonged times in HU (Fig 2B). In summary, we conclude that the SAS-I complex is responsible for the vast majority of DNA replication-coupled deposition of H4 K16Ac, and that H4 K16Ac is incorporated into chromatin at or shortly after replication and chromatin assembly.

### Strong transcription counteracts replication-dependent deposition of H4 K16Ac

**Fig 4:**
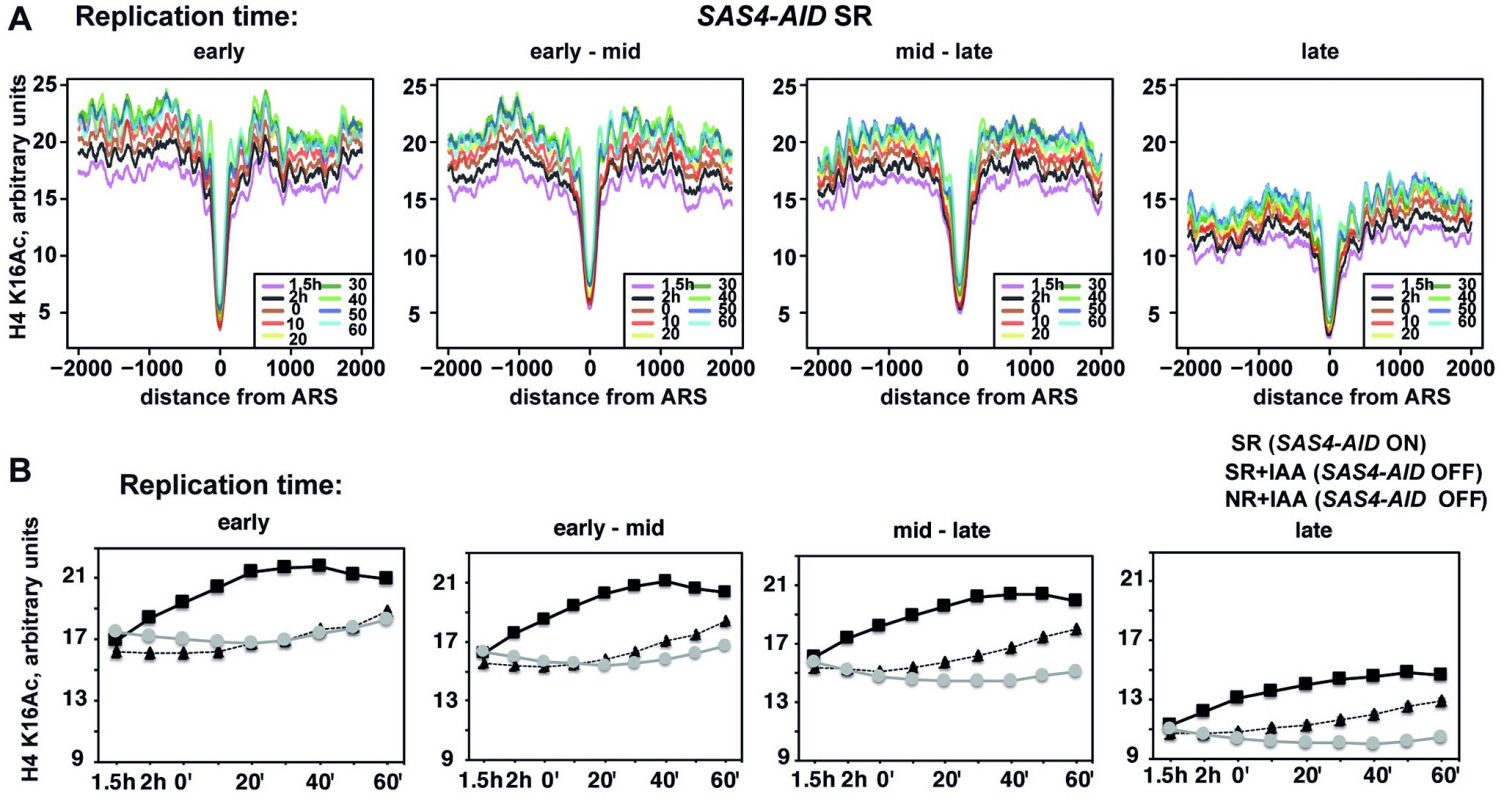
Dynamics of H4 K16Ac on origins of replication during replication. H4 K16Ac increased later on late-replicating origins. (A) Profiles of H4 K16Ac 2 kB upstream and downstream of ARS sequences. Origins were grouped according to their time of replication (from left to right: the earliest 25% of ARS (“early”); second earliest origins (25–50%, “early—mid”); later origins (50–75%, “mid—late”); latest origins (“late”). (B) H4 K16Ac was averaged over the cluster of ARS (ARS +/- 2 kB, but excluding 500 bp surrounding the ARS) and across the time courses.

To further characterize the increase of H4 K16Ac during replication, we generated metaplots of H4 K16Ac levels surrounding replication origins (autonomous replicating sequences, ARS) that were grouped according to their time of replication during S-phase [26]. H4 K16Ac levels were generally higher in earlier replicating regions and lower at later replicating ARS (Fig 4A, S2 Fig). The average increase of H4 K16Ac in the 5 kB region surrounding the ARS (excluding 500 bp nucleosome-depleted region centred on the ARS) occurred earlier at the earlier replicating origins and was not seen in cells depleted of Sas4 with or without release into S-phase (Fig 4B). Of note, the delayed increase of H4 K16Ac in late-replicating origins was not a consequence of this group of origins containing more origins subject to telomeric silencing, because there is no correlation between replication timing and the distance of an origin to its nearest telomere (S2A–S2C Fig).

**Fig 5:**
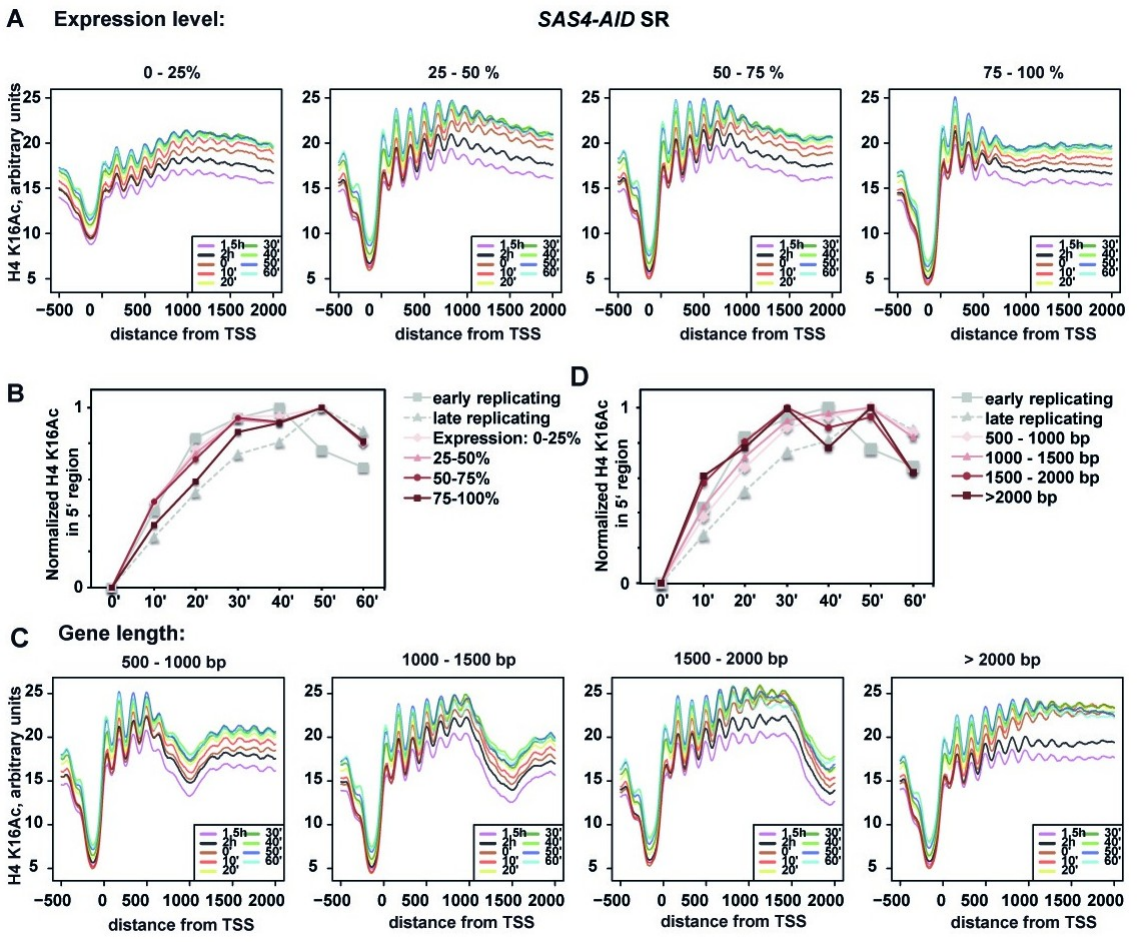
Dynamics of H4 K16Ac on genes of varying expression level. (A) The average H4 K16Ac in the expression clusters is plotted relative to the distance to the transcription start site (TSS). Genes were grouped into clusters based on gene expression in HU-arrested cells (0–25%, lowest expression; 75–100%, highest expression). (B) Genes with high expression showed a delay in replication-mediated increase of H4 K16Ac. H4 K16Ac was averaged across the genes of the cluster, but excluding the first 350 bp. Signals were adjusted to the same dynamic range. (C, D) Replication-associated increase of H4 K16Ac is more prominent in long compared to short genes. (C) Profiles were generated as in A, except that genes were selected by length (500–1000 bp; 1000–1500 bp; 1500–2000 bp; > 2000 bp). (D) Increase of H4 K16Ac during replication occurs earlier on longer compared to shorter genes. Representation as in B.

We were also interested to see the dynamics of H4 K16Ac during DNA replication on expressed genes, since their chromatin is perturbed not only by chromatin disassembly and reassembly during replication, but also by histone turnover during transcription [32]. Metagene plots of genes sorted by expression level showed an increase in H4 K16Ac as cells progressed through S-phase (Fig 5A). Interestingly, this increase followed the dynamics of increase in H4 K16Ac during replication, but only for the genes with the lower 75% of expression level (Fig 5B; expression level 0–25%, 25–50%, 50–75%). Conversely, the genes with the highest expression level (75–100%) showed a delay in the S-phase-dependent increase in H4 K16Ac (Fig 5B). This indicates that strong transcription in these genes counteracts the replication-dependent increase in H4 K16Ac.

Our earlier work has shown that SAS-I-mediated H4 K16Ac is strongest in the 3’ end of longer genes [2]. We therefore investigated the dynamics of H4 K16Ac in dependence of the length on the genes. This showed that the increase in H4 K16Ac was more pronounced at the 3’ end of longer genes than in shorter genes (Fig 5C). That is, the increase of H4 K16Ac was most evident in the 3’ region of genes of 1500–2000 bp length and of genes longer than 2000 bp (Fig 5C, two plots on the right), whereas the increase was less pronounced in the shorter genes (Fig 5C, 500–1000 bp and 1000–1500 bp, two plots on the left). Furthermore, the increase of H4 K16Ac in the longer genes followed the dynamics of H4 K16Ac incorporation in early-replicating genomic regions (Fig 5D, genes > 2000 bp and 1500–2000 bp), whereas the replication-dependent increase of H4 K16Ac occurred at later times in S-phase in shorter genes (Fig 5D, 500–1000 bp, 1000–1500 bp). This indicates that the increase of H4 K16Ac in genes is mostly due to H4 K16Ac deposition during replication, and that it is counteracted by transcription-mediated effects on chromatin near the transcription start site, which thus dominates H4 K16Ac effects in shorter genes as compared to longer genes.

### The absence of both SAS-I and Esa1 abrogates detectable H4 K16Ac

The SAS-I complex is responsible for approx. 60% of bulk H4 K16Ac, indicating that another HAT (or HATs) perform the remaining 40% of H4 K16 acetylation. Esa1 is the catalytic subunit of the NuA4 HAT complex, which has previously been report to acetylate H4 K16 *in vivo*, though to a lesser extent than the SAS-I complex [14–16]. Its major targets are other lysine residues in H4 (K5, K8, K12), and it also acetylates H2A as well as other non-histone proteins [15, 33]. Since it acetylates H4 K16, this makes Esa1 the most likely HAT for the remainder of H4 K16 acetylation in *sas2Δ* strains. To test this, we constructed a double mutant of *sas2Δ* with the temperature-sensitive allele *esa1-L327S* [34]. However, the level of H4 K16Ac in this strain was similar to that of *sas2Δ* even at the restrictive temperature (S3 Fig). We also tested H4 K16Ac levels in double mutants of *sas2Δ* with deletions of the genes encoding Hat1 [35], Sas3 [36], Gcn5 [37], Rtt109 [38, 39], Hpa2 [40] or Spt10 [41]. However, none of the double mutants showed a reduction of H4 K16Ac beyond the level in *sas2Δ* (S3 Fig).

**Fig 6:**
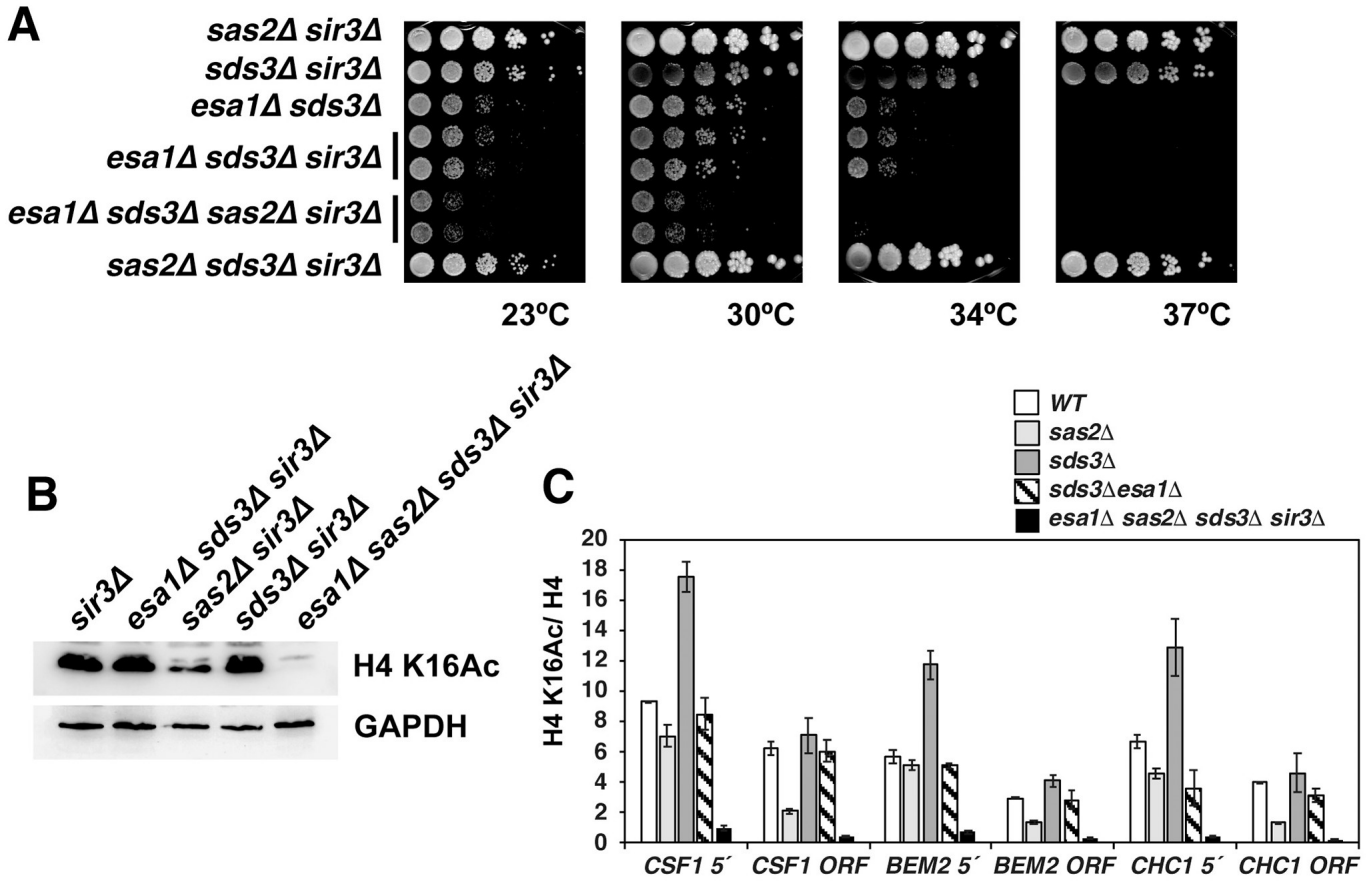
Yeast lacking both SAS-I and Esa1 show no detectable H4 K16Ac. (A) Absence of Sas2 caused a further decrease of viability in *esa1Δ sds3Δ* cells. Strains with the indicated genotypes were serially diluted and spotted on complete medium. Plates were incubated at the indicated temperatures for 2–3 days. Strains were *sir3Δ* in order to circumvent the lethality of *sas2Δ* with *sds3Δ*. H4 K16Ac was undetectable in *esa1Δ sas2Δ* cells by Western blotting (B, as in Fig 1A) and ChIP (C). (C) shows H4 K16Ac relative to H4 (mean ± SD of three independent biological replicates).

Our analysis of the effect of *esa1-L327S* on H4 K16Ac was hampered by the fact that it is a hypomorphic rather than a null allele, which may explain why H4 K16Ac levels are not reduced. We therefore sought for a way of constructing *sas2Δ esa1Δ* double mutants. *ESA1* is essential, but the *esa1Δ* lethality is suppressed by the loss of Sds3, a noncatalytic subunit of the Rpd3L histone deacetylase complex, though *esa1Δ sds3Δ* cells have a strong growth defect [16]. However, a *esa1Δ sds3Δ sas2Δ* strain is expected to be inviable, because *sds3Δ* is synthetically lethal with *sas2Δ* due to the excessive spreading of telomeric heterochromatin into euchromatin regions [10]. That is, the absence of the heterochromatin component Sir3 renders *sas2Δ sds3Δ* strains viable, and accordingly, the *sas2Δ sds3Δ sir3Δ* triple mutants show no growth defect (Fig 6A).

In order to obtain a strain deleted for both *SAS2* and *ESA1*, we therefore generated a *sas2Δ esa1Δ sds3Δ sir3Δ* quadruple mutant (see Materials and methods for details). Whereas *esa1Δ sds3Δ* and *esa1Δ sds3Δ sir3Δ* showed a strong growth defect, the growth of *sas2Δ esa1Δ sds3Δ sir3Δ* was even more defective (Fig 6A), indicating that the combined loss of acetylation targets of SAS-I and NuA4 is detrimental to the cell. Of note, *sir3Δ* did not improve the growth of *esa1Δ sds3Δ*, although previous reports had shown that the absence of Sir2, the deacetylase subunit of the SIR heterochromatin complex, partially suppressed the growth defect of *esa1Δ sds3Δ* [16]. This indicated that the improved growth of *esa1Δ sds3Δ sir2Δ* was not caused by the loss of SIR-mediated silencing, and it thus may be due to increased acetylation of non-histone targets of Sir2.

We next tested H4 K16Ac levels in the *sas2Δ esa1Δ sds3Δ sir3Δ* strain by Western blotting and ChIP. As expected, bulk H4 K16Ac was reduced in *sas2Δ* as compared to wt (Fig 6B), and ChIP also showed a reduction at the genes *CSF1*, *BEM2* and *CHC1*, both at the 5’ end and within the open reading frame (ORF) (Fig 6C). The level in *sds3Δ*, which lacks the Rpd3L HDAC, was similar to that in wt in bulk H4 K16Ac and was either similar to or increased compared to wt by ChIP. Interestingly, the additional deletion of *ESA1* in *sds3Δ* decreased H4 K16Ac levels at the 5’ ends of the genes tested compared *sds3Δ*, showing that *esa1Δ* causes a decrease of H4 K16Ac. Importantly, we were unable to detect any H4 K16Ac by Western blotting (Fig 6B) nor by ChIP (Fig 6C) in *sas2Δ esa1Δ sds3Δ sir3Δ* cells. This showed that the majority, if not all of H4 K16Ac that is present in *esa1Δ sds3Δ* was mediated by the SAS-I complex, thus suggesting that Esa1 and SAS-I are the sole HATs for H4 K16 in *S*. *cerevisiae*.

## Discussion

During DNA replication, nucleosomes are disassembled in front of the replication fork, and H3/ H4 is distributed onto the two daughter strands. To provide the full histone complement, newly synthesized H3/ H4 is incorporated into the new chromatin by CAF-I and Asf1 [11]. H4 K16Ac is found predominantly in the body of genes with low transcription, and it is required to prevent the spreading of heterochromatin into subtelomeric regions [2]. It is therefore important that H4 K16Ac patterns are restored after DNA replication and chromatin assembly.

Here, we investigated the dynamics of H4 K16Ac during DNA replication. We found H4 K16Ac to increase immediately at the time of replication, a finding that is in agreement with earlier work [25]. Moreover, this replication-dependent increase of H4 K16Ac depended on the presence of Sas4, a component of the SAS-I HAT complex [3, 4]. Since the SAS-I complex interacts with the chromatin assembly factors CAF-I and Asf1 [3, 4], this observation supports the notion that SAS-I is recruited to newly assembled chromatin to acetylate H4 on K16. Furthermore, the replication-dependent increase of H4 K16Ac occurred earlier in genes that are lowly transcribed and later in strongly expressed genes. We propose that the steady-state genome-wide level of H4 K16Ac is the result of acetylation by SAS-I in the wake of replication-coupled chromatin assembly, which then is sculpted by transcription-coupled histone exchange within the coding region of genes. This underscores the notion that SAS-I acetylates new histones at the time of their deposition into chromatin by CAF-I and Asf1. One possibility is that SAS-I is recruited to chromatin by its interaction to CAF-I. Alternatively, SAS-I may acetylate the histones as they are bound by CAF-I/ Asf1, but shortly before incorporation into chromatin.

We furthermore found that the vast majority of H4 K16Ac in *S*. *cerevisiae* is performed redundantly by SAS-I and Esa1, a component of the NuA4 and piccolo NuA4 complexes. In order to obtain viable *esa1Δ sas2Δ* strains, the cells were also rendered defective for the HDAC complex Rpd3L (to rescue the lethality of *esa1Δ*) and for the heterochromatin complex SIR (to suppress the lethality of *sas2Δ sds3Δ*). The observation that *sas2Δ esa1Δ sds3Δ sir3Δ* cells have an even stronger growth defect than *esa1Δ sds3Δ sir3Δ* cells indicates that SAS-I-mediated acetylation has a cellular function that extends beyond its function in preventing the spreading of the SIR complex.

## Supporting information

S1 Fig Genome-wide analysis of H4 K16Ac in cells depleted for Sas4-AID and arrested in S-phase. There was only a marginal increase of H4 K16Ac at late experimental timepoints. Representation as in Fig 3.
(TIF)

S2 Fig Replication timing of genomic features in yeast. (A) Grouping of yeast replication origins according to their replication timing as determined previously [42]. (B) shows the distance of the yeast ARS sequences as grouped in (A) to their nearest telomere. (C) Plot of the distance of an ARS from its telomere relative to its time of replication. (D) Replication time (in minutes) of yeast genes grouped by their length (bp). (E) Plot of gene length relative to the time of replication.
(TIF)

S3 Fig H4 K16Ac levels in yeast strains lacking Sas2 and other histone acetyltransferases. Whole cell extracts of the indicated strains were analyzed by PAGE and Western blotting with α-H4 K16Ac (top) and α-H2B (bottom, loading control). H4 K16R, strain carrying H4 with a mutation of lysine 16 to arginine (control for antibody specificity).
(TIF)

S1 Raw images (PDF)
