# Supplementary figures and images for: Dynamics of SAS-I mediated H4 K16 acetylation during DNA replication in yeast
Source: PLoS One. 2021 May 20;16(5):e0251660. doi: 10.1371/journal.pone.0251660 (PMC8136709; doi:10.1371/journal.pone.0251660)

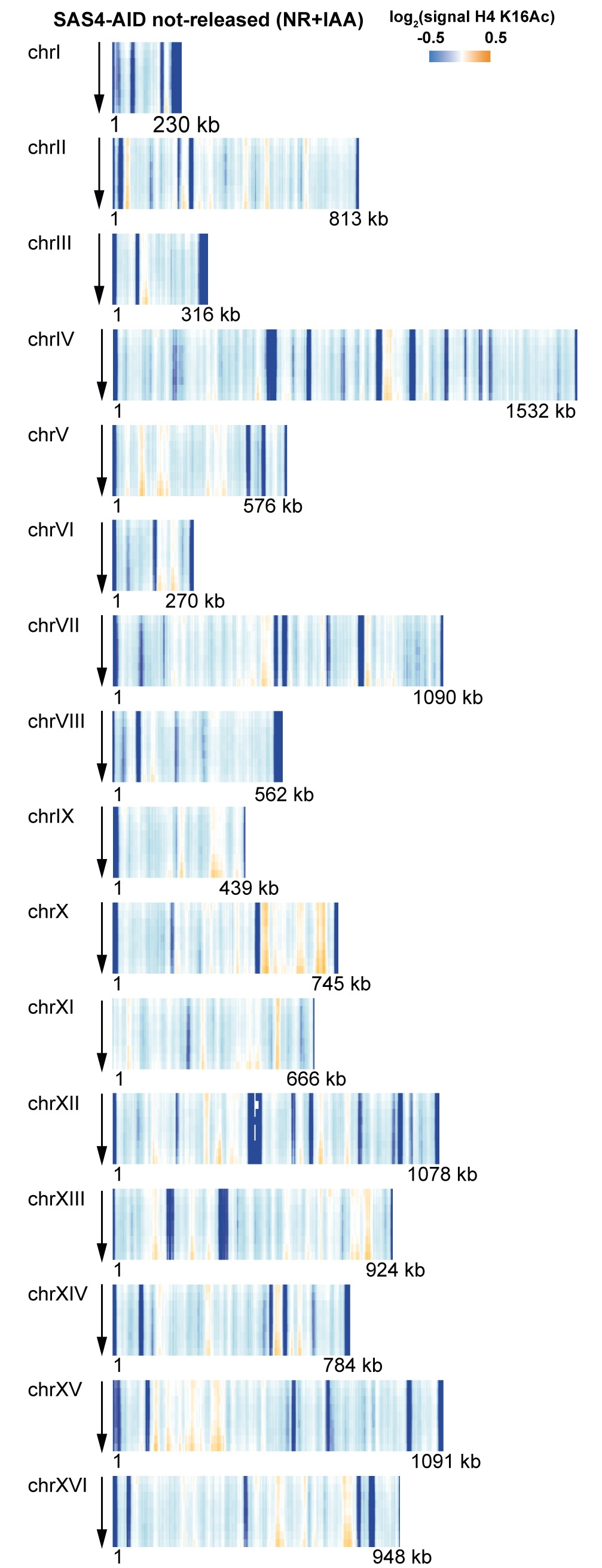

Supplement: S1 Fig — There was only a marginal increase of H4 K16Ac at late experimental timepoints. Representation as in Fig 3. (TIF) [file pone.0251660.s001.tif]

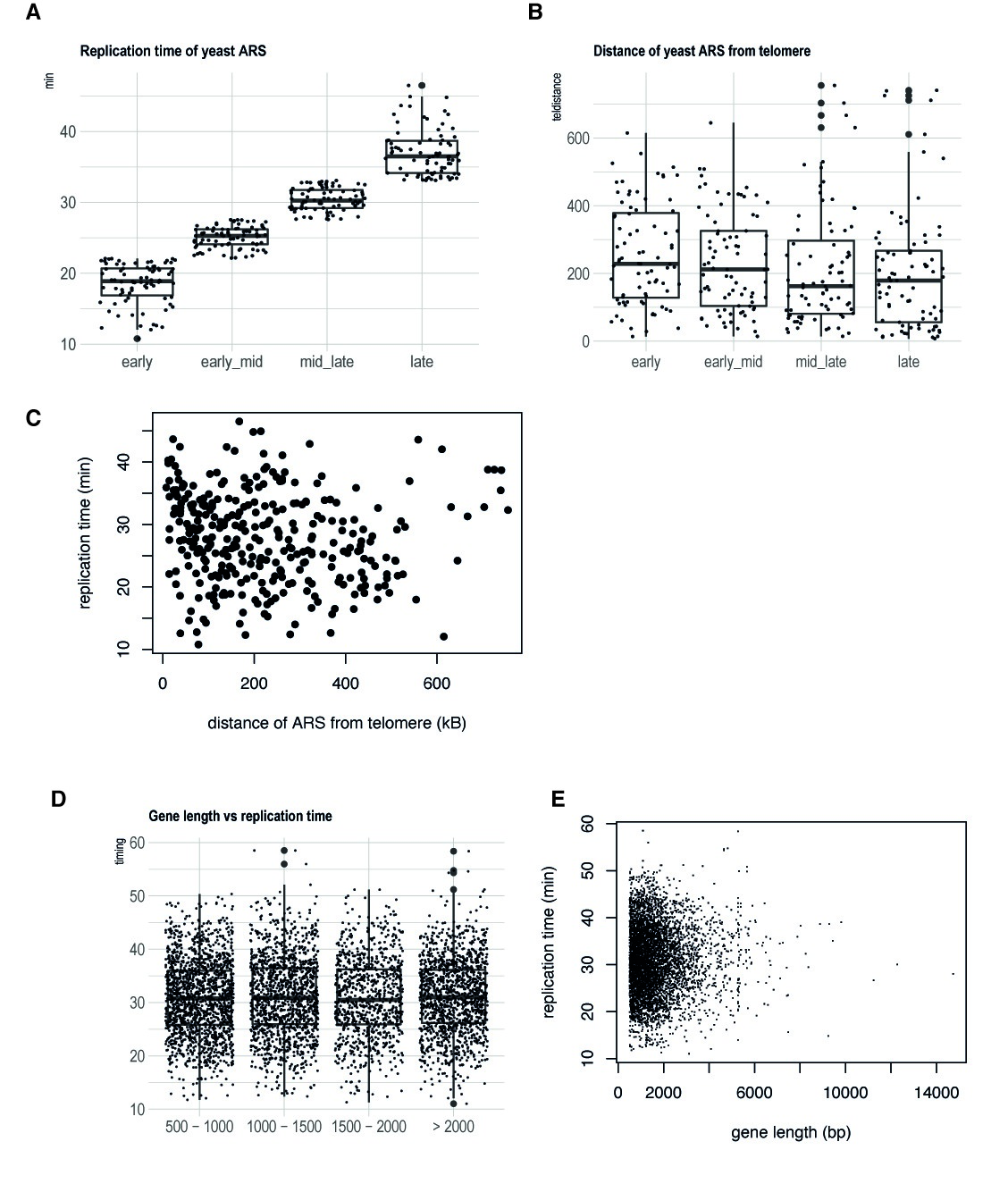

Supplement: S2 Fig — (A) Grouping of yeast replication origins according to their replication timing as determined previously [42]. (B) shows the distance of the yeast ARS sequences as grouped in (A) to their nearest telomere. (C) Plot of the distance of an ARS from its telomere relative to its time of replication. (D) Replication time (in minutes) of yeast genes grouped by their length (bp). (E) Plot of gene length relative to the time of replication. (TIF) [file pone.0251660.s002.tif]

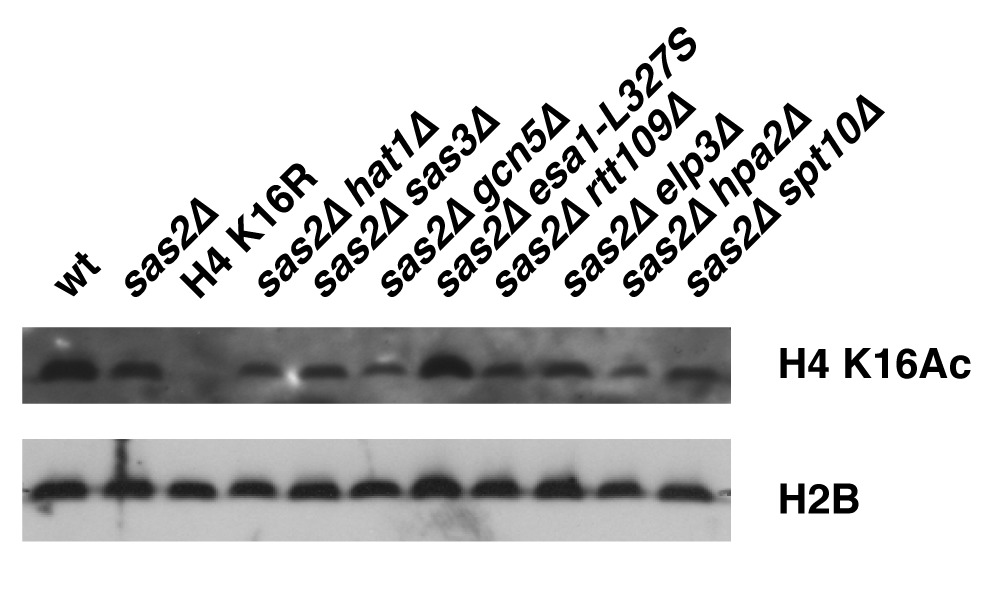

Supplement: S3 Fig — Whole cell extracts of the indicated strains were analyzed by PAGE and Western blotting with α-H4 K16Ac (top) and α-H2B (bottom, loading control). H4 K16R, strain carrying H4 with a mutation of lysine 16 to arginine (control for antibody specificity). (TIF) [file pone.0251660.s003.tif]

Fig. 1A

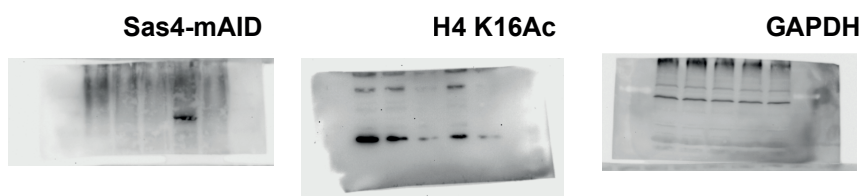

Fig. 2C

SAS4-AID SR+IAA

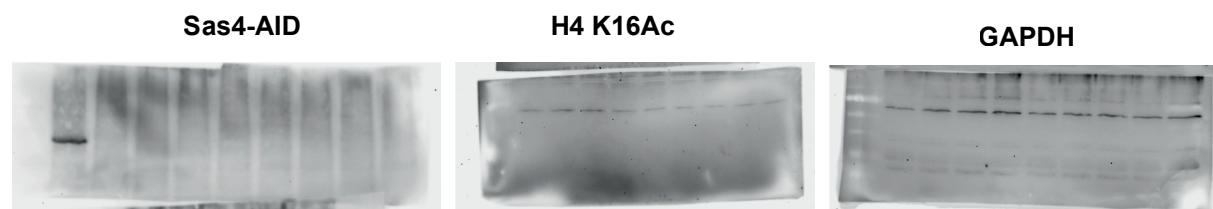

SAS4-AID SR

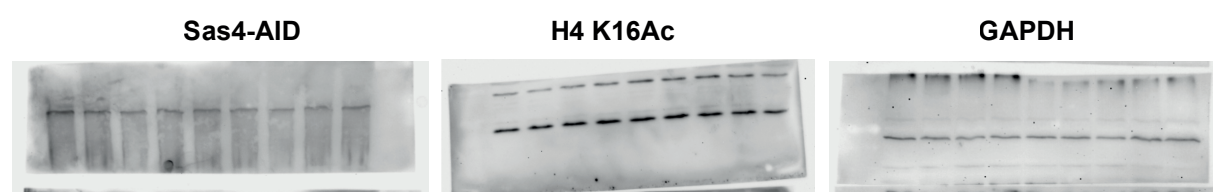

SAS4-AID NR+IAA

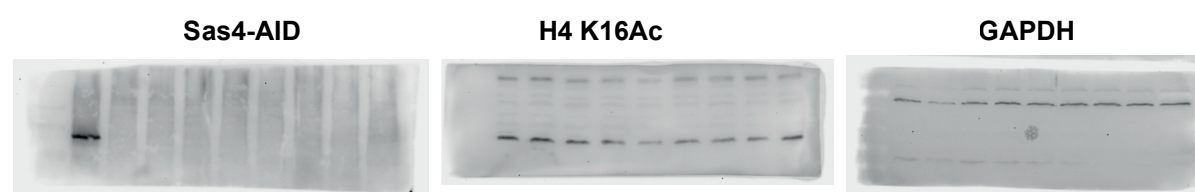

Fig. 6B

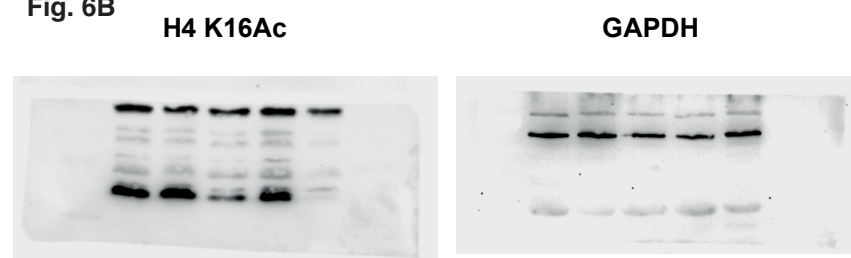

Fig. S3

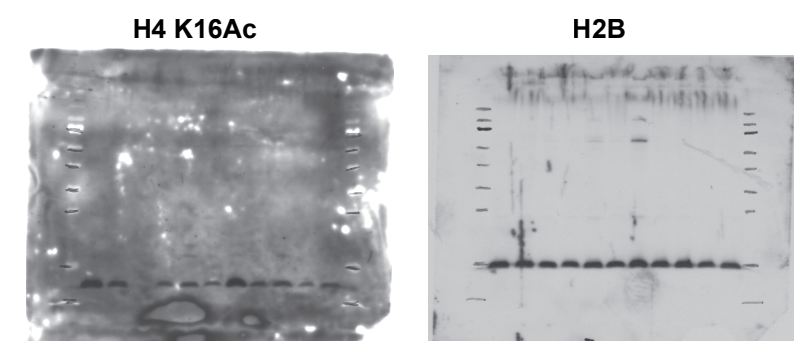

Supplement: S1 Raw images — (PDF) [file pone.0251660.s004.pdf]
