# Supplementary material for: Dynamics of SAS-I mediated H4 K16 acetylation during DNA replication in yeast
Source: PLoS One. 2021 May 20;16(5):e0251660. doi: 10.1371/journal.pone.0251660 (PMC8136709; doi:10.1371/journal.pone.0251660)
Supplement: Attachment — Submitted filename: Rebuttal_PlosOne_230321..docx [file pone.0251660.s005.docx]

Humboldt-Universität zu Berlin | Unter den Linden 6 | 10099 Berlin

Plos One

Datum:

23.3.2021

Bearbeiter/in:

Geschäftszeichen:

Dear editors, dear reviewers,

I am herewith submitting a revision of our manuscript entitled „ **Dynamics of SAS-I mediated H4 K16 acetylation during DNA replication in yeast**“ for consideration at Plos One.

Former submission ID: **PONE-D-20-02416**

We had originally submitted this manuscript in January 2020 for review and received the reviewer comments on Feb. 20, 2020. Unfortunately, the Corona pandemic struck while we were working on the revision, and the first author, Mark Boltengagen, left the lab. We had to close the lab for several months. Once we were able to reopen, the experiments were taken over by another postdoc from my lab, Anke Samel-Pommerencke (now added as an author). We were therefore severely delayed in performing the additional experiments requested by the reviewers and could not meet the deadlines that were set (and extended several times). The manuscript was withdrawn from the Plos One system on Dec. 11, 2020.

We now provide the revision and ask you to consider it for publication in Plos One.

In the revision, we have addressed all points raised by the reviewers as well as the editor. The original conclusions remain unchanged by the new experiments. We hope that the work now is acceptable for publication in Plos One. A point-by-point response is attached.

Please note that we also are providing all raw blot images (file S1_raw_images). Furthermore, we are providing one file with all changes marked in red and an unmarked version of the manuscript.

Many thanks in advance for your time and consideration, and I look forward to hearing from you.

Sincerely,

Ann Ehrenhofer-Murray

Point-by-point response to reviewer comments:

Reviewer #1:
*1. Figure 2 – If logarithmic cells are exposed to hydroxyurea, they will arrest throughout S phase (early, mid, late + cells in G2/M, G1 will arrest following early S), not just at “early S phase” Were cells pre-synchronized prior to adding HU in this experiment? It is unclear where the “mid- and late-S phase-arrested” population is and the effects on the patterns in Fig. 2 D.*

The protocol that we followed is like in Bar-Ziv et al (PMID 27225843) to allow comparison with that study. Operationally, hydroxyurea was added to the cells in order to arrest them in S-phase. The phrase “early S-phase” has been removed from the text.

*2. Figure 2 legend and figure - The variations in labeling and names in the current form is very confusing. Redefine SR-IAA, HU-IAA (is this what is called NR-IAA in panels A and B and SAS4-AID NR in panel C?), and SR, and use a single nomenclature to refer to each condition in the figure panels, figure legend and text. The labels change yet again in panel D. And change yet again in later panels, figure legends (see below). (I don’t care which form you pick, just use one throughout the entire manuscript to refer to an individual sample/condition, and define it, don’t use a bunch of different names for the same sample).*

We apologize for the confusion. The labelling has been made more intuitive (SR+IAA, SR, NR+IAA) and has been unified throughout the ms.

*3. For Figure 2D. The second and third panel are labeled identically and labels do not match other panels in figure. This needs to be corrected. For Figure 2D, what do the top two rows in each panel represent? Please clarify in labels on Figure and in legend.*

The labelling has been adjusted. The second and third panel represent synchronized, IAA-treated, released (SR+IAA) and synchronized, IAA-treated, non-released (NR+IAA) samples.

*4. Please include positions of all chromosome XI origins into Figure 2D on the x axes. Currently it is not clear whether H4 K16ac pattern is detected at sites flanking early origins only, or also at mid and late S phase-firing origins that should have fired and be flanked by HU-arrested forks in a subpopulation of the arrested cells (see point 1 above).*

Many thanks for the suggestion. We have addressed this point by including a panel in Fig. 2D that shows the replication profile for chromosome XI as determined by Raghuraman et al (Science 2001). This has the advantage over simply showing the location of the ARS sequences that it also shows whether the origins (peaks) are early or late firing.

*5. Please clarify how or whether unreplicated versus replicated DNA is being controlled for the ChIPseq data timecourse. Is the increase in signal due to twice as much DNA being available to IP, or more H4 K16ac being present after normalizing for DNA content?*

Our analysis was performed as in Bar-Ziv et al (2016) and described in Materials and Methods. In brief, the abundance of H4 K16Ac was calculated along the yeast chromosomes relative to the average in each time-course. Normalization at an individual time-point was done by log_2_-transforming the 15-kB bin average (moving average 2 kB) and then subtracting the average signal of the bin along the time-course (c.f. Bar-Ziv 2016, Fig. 1F). We did not normalize for DNA content. The increase of H4 K16Ac must exceed the DNA content, because the increase is largely abrogated when Sas4 is switched off before releasing the HU-arrested cells (Fig. 2D, SR+IAA).

*6. Points 1-5 should also be clarified for Figure 3 and Figure 3 legend (and Figure S1 and S1 legend). Labels should match nomenclature for Figure 2 to make graphics and text consistent throughout manuscript to avoid confusion.*

Labels have been adjusted.

*7. Labels in Figure 4 should also match nomenclature for Figure 2, 3, S1 to make graphics and text consistent throughout manuscript to avoid confusion.*

Labelling in Figure 4 has been adjusted.

*8. For Figure 4A,B, late origins, could the reduced signal be, in part, a reflection of a small fraction of cells having originally arrested in late S phase? See point 1. Or is this a reflection of late origins being enriched at telomeres, where Sir2p (and Hsts?) would be expected to deacetylate H4 K16, thereby leading to the reduced H4 K16ac signal by the late origins? Correlation analyses would help authors and readers understand why the patterns being observed are being observed.*

We investigated whether the low H4 K16Ac at the late origins could be a consequence of subtelomeric silencing by the SIR complex. However, there was no correlation between replication time and distance to the telomere, and the average distance of the late-replicating ARS from the telomere was not shorter than e.g. the group of mid-late replicating origins. We have added this information to the text of the ms and have generated a new suppl. figure (Fig. S2) to show the analysis.

*9.Does the dip in H4 K16a at the 3’ end of genes in Fig. 4E reflect the promoter of the next gene? How was this data normalized? Presumably, it has not been normalized to another histone, so the dip may be the signal at the TSS is a nucleosome Free region. Correlation analyses would help authors and readers understand why the patterns being observed are being observed.*

We investigated whether the dip at the 3’ end of genes could reflect the promoter of the next gene by investigating H4 K16Ac in intergenic regions of convergent genes (i.e. these regions contain no promoter). This showed that there was an increase of H4 K16Ac over the time course at the intergenic convergent regions that depended on Sas4, whereas the slight increase observed at promoters was independent of Sas4 (Reviewer fig. below). We therefore conclude that the dip of H4 K16Ac at the 3’ end of genes is a characteristic of the 3’ end, and does not reflect the NFR of the “next” gene.

Reviewer figure:

Analysis of H4 K16Ac in SAS4-AID at intergenic regions of convergent genes and at promoters

Figure legend: Low levels of H4 K16Ac at the 3’ end of genes is not a reflection of the nucleosome-free region of the next gene downstream. H4 K16Ac was averaged over intergenic regions of convergent genes (left) or over the promoter of all genes (right) over the time course of the experiment. Representation as in Fig. 4B.

*10. Figure 4F the authors state that the increase in H4 K16ac during replication occurs earlier on longer compared to earlier genes. What have the authors done to assess why this is? For example are longer genes enriched by early origins?*

To investigate this question, we looked at the replication time of genes relative to their length. However, there was no correlation between gene length and replication time, and the group of long genes (> 2000 bp) showed a similar distribution of replication times compared to the other gene groups. We have added this information to the ms text and provide the respective plots in the new supplementary figure S2.

11. Line 267, “…genes longer than 200 bp…” do you mean “2000 bp”?

*Sorry for the mistake, it has been corrected.*

12. In Materials and Methods, please clarify how the RNAseq data was analyzed. Are expression levels dependent on or independent of Sas4?

*The RNA-seq data was analysed using the Salmon tool, and transcripts were sorted by transcription level. This information has been added to M + M. We did not test dependence on Sas4.*

13. Figure 5C, state how the ChIP data was analyzed in the legend or the Materials and Methods. If the authors have not normalized to histone content at the locus of interest, how do they know any observed reduction (or increase) in signal isn’t due to reduced (or increased) nucleosome levels at the locus of interest? How do the mutants affect transcription at these loci? Could this influence total H4 at these loci, thereby affecting H4 K16ac signal and the interpretation of the data?

*We have repeated the ChIP experiment from this Figure (now Fig. 6C) and have normalized H4 K16Ac to H4. The experiment confirms our finding that H4 K16Ac is very low/ undetectable in cells lacking both Esa1 and Sas2.*

14. Line 362-363. The statement “…all detectible H4 K16ac in S. cerevisiae is performed redundantly by SAS-I and Esa1,…” is an overinterpretation of the data provided in this manuscript and should be removed accordingly.

*We have weakened the statement in order to not overinterpret the data.*

Reviewer #2:

1. Currently the abstract is extremely long and can be shortened. In particular, the final section could omit the genetic details and simply state the conclusion.

*We have shortened the final section of the abstract.*

2. The authors might wish to comment why they decided to synchronize cells in early S phase with HU, rather than using a more common method to use alpha factor to arrest in G1. Their method does not detract from the conclusions, but it may be worth a comment.

*We used HU, because we wanted to be able to compare our results to those of Bar-Ziv et al (PMID 27225843), and we therefore adopted the method from them.*

3. Figure 2B. The asynchronous FACS profiles seem quite odd, with a large peak of G2 cells, rather than the normal enrichment of G1 cells. The authors should comment.

*We saw similar profiles across several experiments (different days, different time courses) and therefore did not think much of it.*

4. Figure 2D. The term "sync" should be defined in the figure legend. I assume this means HU arrest?

*Yes, “sync” means synchronization (i.e. HU-arrest). The explanation has been added to the figure legend.*

5. Figure 2D. There seems to be a large genomic region at around 550 kb where H4K16Ac levels stay high even in the absence of Sas4. Does this region contain an interesting locus? Likewise, in many of the ChIP-seq figures, there are regions of intense blue (depletion). Are these non-mapping regions or regions that do not have K16Ac (e.g. telomeres or centromeres)?

*This region contains the DYN1 gene, which is the second longest gene in the yeast genome (12,3 kB). This explains why it has a relatively high level of H4 K16Ac (compared to other regions of chrXI). It loses some of H4 K16Ac when Sas4 is switched off, but gains some during replication.*

Additional changes to the ms:

- In addition to the requests for the reviewers, we have added a new supplementary figure 3 (Fig. S3) that shows the levels of H4 K16Ac in *sas2∆* strains with deletions/ mutations in additional HATs. This was previously mentioned as “data not shown” in the ms.

- Original Fig. 4 was split into two figures (Fig. 4 and Fig. 5) in order to meet the formatting requirements of Plos One. Former Fig. 5 is now Fig. 6.

- We have repeated the ChIP experiment in Fig. 1C, which measures H4 K16Ac levels upon Sas4 depletion. The ChIP levels shown now are normalized to H4.

- We have repeated the analysis of repression of Sas4-AID by addition of auxin (Fig. 1B), which shows that Sas4-AID provides silencing function in the absence of auxin, and the silencing function is lost upon addition of auxin.
