# Supplementary material for: Dynamics of SAS-I mediated H4 K16 acetylation during DNA replication in yeast
Source: PLoS One. 2021 May 20;16(5):e0251660. doi: 10.1371/journal.pone.0251660 (PMC8136709; doi:10.1371/journal.pone.0251660)
Supplement: Peer review history

# Author comment: Author response to previous submission

**DOI:** 10.1371/journal.pone.0251660.r001

23 Mar 2021

Attachment Submitted filename: Rebuttal_PlosOne_230321..docx Click here for additional data file.

# Editor report: Decision Letter 0

**DOI:** 10.1371/journal.pone.0251660.r002

**Authors:** Mary Bryk

30 Apr 2021

Dynamics of SAS-I mediated H4 K16 acetylation during DNA replication in yeast

PONE-D-21-09570

Dear Dr. Ehrenhofer-Murray,

We’re pleased to inform you that your manuscript has been judged scientifically suitable for publication and will be formally accepted for publication once it meets all outstanding technical requirements.

Within one week, you’ll receive an e-mail detailing the required amendments. When these have been addressed, you’ll receive a formal acceptance letter and your manuscript will be scheduled for publication.

An invoice for payment will follow shortly after the formal acceptance. To ensure an efficient process, please log into Editorial Manager at http://www.editorialmanager.com/pone/, click the 'Update My Information' link at the top of the page, and double check that your user information is up-to-date. If you have any billing related questions, please contact our Author Billing department directly at authorbilling@plos.org.

If your institution or institutions have a press office, please notify them about your upcoming paper to help maximize its impact. If they’ll be preparing press materials, please inform our press team as soon as possible -- no later than 48 hours after receiving the formal acceptance. Your manuscript will remain under strict press embargo until 2 pm Eastern Time on the date of publication. For more information, please contact onepress@plos.org.

Kind regards,

Mary Bryk, Ph.D.

Academic Editor

PLOS ONE

Additional Editor Comments:

This manuscript was submitted previously and had a full set of reviews from the first round. The authors have addressed the reviewers' comments and added data that strengthened the findings reported in the manuscript. I have a few minor comments.

Page 13 line 266 buffer is misspelled, puffer.

Page 19 Figure 4B. Your point would be easier to see if the graphs had the same y axis.

Page 21. Figure 5D. Less H4K16Ac at 3' ends of 1000-1500 bp genes is difficult to see.

Page 23 Figure 6C. The key for this graph is too small.

Page 23 line 518 What is the evidence that the increased acetylation must have been on non-histone targets of Sir2?  Sir2 works at genomic locations other than telomeres. Consider toning down this statement.

# Editor report: Acceptance letter

**DOI:** 10.1371/journal.pone.0251660.r003

**Authors:** Mary Bryk

11 May 2021

PONE-D-21-09570 

Dynamics of SAS-I mediated H4 K16 acetylation during DNA replication in yeast 

Dear Dr. Ehrenhofer-Murray:

I'm pleased to inform you that your manuscript has been deemed suitable for publication in PLOS ONE. Congratulations! Your manuscript is now with our production department. 

If your institution or institutions have a press office, please let them know about your upcoming paper now to help maximize its impact. If they'll be preparing press materials, please inform our press team within the next 48 hours. Your manuscript will remain under strict press embargo until 2 pm Eastern Time on the date of publication. For more information please contact onepress@plos.org.

If we can help with anything else, please email us at plosone@plos.org. 

Thank you for submitting your work to PLOS ONE and supporting open access. 

Kind regards, 

PLOS ONE Editorial Office Staff

on behalf of

Dr. Mary Bryk 

Academic Editor

PLOS ONE
